# The oncogenic role of hepatitis B virus X gene in hepatocarcinogenesis: recent updates

**DOI:** 10.37349/etat.2024.00209

**Published:** 2024-02-20

**Authors:** Agustiningsih Agustiningsih, Muhammad Rezki Rasyak, Turyadi, Sri Jayanti, Caecilia Sukowati

**Affiliations:** The First Clinical Medical College of Lanzhou University, China; ^1^Eijkman Research Center for Molecular Biology, National Research and Innovation Agency of Indonesia (BRIN), Jakarta Pusat 10340, Indonesia; ^2^Post Graduate School, Faculty of Medicine, Universitas Hasanuddin, Makassar 90245, Indonesia; ^3^Liver Cancer Unit, Fondazione Italiana Fegato ONLUS, AREA Science Park, Basovizza, 34149 Trieste, Italy

**Keywords:** Hepatitis B virus, hepatitis B virus X protein, hepatocellular carcinoma, signalling pathways

## Abstract

Hepatocellular carcinoma (HCC) is the most prevalent form of primary liver cancers with high mortality rate. Among its various etiological factors, one of the major risk factors for HCC is a chronic infection of hepatitis B virus (HBV). HBV X protein (HBx) has been identified to play an important role in the HBV-induced HCC pathogenesis since it may interfere with several key regulators of many cellular processes. HBx localization within the cells may be beneficial to HBx multiple functions at different phases of HBV infection and associated hepatocarcinogenesis. HBx as a regulatory protein modulates cellular transcription, molecular signal transduction, cell cycle, apoptosis, autophagy, protein degradation pathways, and host genetic stability via interaction with various factors, including its association with various non-coding RNAs. A better understanding on the regulatory mechanism of HBx on various characteristics of HCC would provide an overall picture of HBV-associated HCC. This article addresses recent data on HBx role in the HBV-associated hepatocarcinogenesis.

## Introduction

Approximately more than 2 billion people in the world are estimated to be affected by hepatitis B virus (HBV) infection. Accordingly, up to 300 million people are suffering with chronic liver disease due to HBV infection. HBV-associated prolonged chronic inflammation and hepatic damage associated would often progress to severe liver diseases including fibrosis, cirrhosis, and eventually hepatocellular carcinoma (HCC) [1, 2]. HCC is the fourth commonest type of human cancers, and currently the third leading cause of death worldwide [3].

HBV is the smallest DNA virus of the *Hepadnaviridae* family of viruses. HBV has a 3.2 kilobases circular and partially double-stranded genome. There are four overlapping open reading frames (ORFs) in HBV genome: P, S, C, and X [4]. The four ORFs encode for seven proteins: pre-S1, pre-S2, S, pre-C, C, viral polymerase, and HBV X protein (HBx) protein; and four regulatory elements: enhancer II/basal core promoter, pre-S1 promoter, pre-S2/S promoter, and enhancer I/X promoter [5]. Using the variation within the viral nucleotide (nt) sequences, nine HBV genotypes (A to I) have been identified, based on an intergroup divergence of more than 7.5% across the genome [6]. In addition, the tenth genotype (J) had been identified from a Japanese individual on the island of Borneo [7, 8].

HBV chronic infection is a strong etiological factor in promoting HCC development. In comparison with other HCC etiological factors such as hepatitis C virus (HCV), alcohol consumption, diabetes, and aflatoxin B1, the onset of HBV-related HCC often develops 10 years earlier. HBV-related HCC also exhibits a higher level of α-fetoprotein (AFP) and more observable microvascular invasion [9]. The molecular mechanisms driving HBV-induced HCC are complex. Several cellular mechanisms have been proposed as the underlying main factors in the occurrence and progression of liver tumorigenesis including viral genome integration into the host genome during viral replication, chronic inflammation induced by the host immune response, and HBx-induced dysregulation of cellular signal transduction pathways [10].

## HBx gene and protein

As the smallest HBV genome ORF, *HBx* gene encodes a 154-amino acid (AA) regulatory protein with molecular weight of 17 kDa. Typically, as observed in HBV genotype B (HBV/B) and genotype C (HBV/C), HBx is located at nt position 1,060-1,838, with HBx promoter region at nt 1,060 to 1,373 and HBx coding region at nt 1,374 to 1,835 [11].

HBx protein is distinct from other proteins because its AA sequence is different to any of the existing protein. HBx protein has two functional domains, the amino-terminal domain (AA 1–50) that may inhibit HBx activities and the trans-activation function domain (AA 52–148) that regulate HBx interaction with other viral and/or host proteins. The trans-activation function domain comprised of three further subdomains with different roles: the signal transduction activities (AA 58–119), the nuclear trans-activation mechanisms (AA 120–140), and the last 20 AA at the C-terminal which is important for HBx stability [12].

HBx is a multifunctional protein that modulates several cellular processes via direct or indirect interaction with various host factors that contributes to viral persistence and disease progression. HBx plays a crucial role in the HBV-related progression of HCC, since HBx may interfere with crucial cellular processes that promotes and contributes to tumorigenesis including signal transduction in the cells, oxidative stress, apoptosis and DNA repair, transcription, protein degradation, and cell cycle progression [10].

## HBx role in hepatocarcinogenesis

### Signal transduction and cell cycle progression

Intracellular localization study using liver biopsy samples from HBV patients showed that highly expressed HBx localizes predominantly in the cytoplasm, whereas lowly expressed HBx localized primarily in the nucleus instead [13, 14]. HBx one of viral factors that strongly triggers HCC carcinogenesis, as suggested by its significantly higher expression in HBV-related HCC as compared to the first phase of viral infection [15, 16]. HBx localization in the cytoplasm of cells, enable it to interact with and modulate various signal transduction pathways of the cells including mitogen-activated protein kinase (MAPK), Src-dependent phosphatidylinositol 3-kinase (PI3K)/protein kinase B (AKT), kinase C signalling cascades, rat sarcoma virus (RAS), rapidly accelerated fibrosarcoma (RAF), Janus kinase-signal transducer and activator of transcription (JAK-STAT), nuclear factor kappa-light-chain-enhancer of activated B cells (NF-κB), and focal adhesion kinase [17, 18]. The transactivation of these cellular signaling molecules can all lead to more proliferation of hepatocytes (Table 1).

**Table 1:** Influence of HBx in cellular pathways and process

**Pathway and process**	**Effect**	**References**
MAPK signaling pathway	Enhancement of MAPK activation leading to cell proliferation, prevention of apoptosis, and gene expression regulation.	[19, 20]
PI3K/AKT signaling pathway	Stimulation of the PI3K/AKT signaling pathway, resulting in apoptosis suppression, increased cell proliferation that might disrupt cellular communication, and boost its survival within host hepatocytes.	[21, 22]
JAK-STAT signaling pathway	Disruption of JAK-STAT signaling system, resulting in immunological suppression, increased cell survival, and proliferation. Interference with the JAK-STAT pathway leads to HBV infection persistence.	[18]
NF-κB signaling pathway	Activation of NF-κB signaling pathway, resulting in enhanced inflammation, immunological regulation, cell survival, and proliferation.	[23, 24]
Oxidative stress	Imbalance between ROS production and antioxidant defense mechanisms within hepatocytes, leading to increased oxidative stress. This oxidative stress contributes to DNA damage, genomic instability, inflammation, and cell survival pathways that collectively promote liver damage.	[25, 26]
Apoptosis and DNA repair mechanism	Inhibition of apoptosis and DNA repair mechanisms in a way that promotes cell survival, inhibits programmed cell death, and can contribute to genomic instability.	[27, 28]
Autophagy mechanism	The effect of HBx-induced autophagy (activation of P13K/AKT-mTOR, and interference with autophagic flow) may help infected hepatocytes survive by eliminating damaged cellular components and promoting viral replication.	[29, 30]
Epigenetic modification	Induction of epigenetic modifications such as DNA methylation, histone alterations, remodeling of chromatin, and various miRNAs that all together affect gene expression patterns and cellular responses.	[31–33]

mTOR: mammalian target of rapamycin; miRNAs: microRNAs

c-Src is a one of the key mediators of HBx-induced dysregulated signaling pathways. A study by Yang et al. [34] in SMMC-7721 cell line suggested HBx-induced epithelial-to-mesenchymal transition (EMT) through activation of c-Src, which led to malignant tumor invasion. Furthermore, HBx binds to the promoters of several host cellular genes, and acts as their transcription factor to promote their expressions. These include AFP and AFP receptor (AFPR) in liver cells, which are tumor development associated genes. AFPR activation leads to activation of the PI3K/AKT signaling pathway, which in turn promotes Src expression and contributes to HBV-associated tumor development [35]. In the oval cells, HBx promoted cellular proliferation. HBx stimulatory effects and cell cycle activity via cyclin D1 were associated with the activation of MAPK kinase (MEK)/extracellularly regulated kinases (ERK) and PI3K/AKT signaling pathways [21, 22].

A study in zebrafish found that HBx-induced activation of Src tyrosine kinase pathways and p53 mutations are associated with hepatocarcinogenesis. This HBx-induced tumorigenesis was only observed in the p53 mutant fish, and was found correlated with activation and upregulation of Src tyrosine kinase pathway [36].

The NF-κB pathway is also dysregulated in HBV-related HCC. In both *in vitro* and *in vivo* studies, HBx induces upregulation and phosphorylation of subunit NF-κBp65. Consistently, NF-κBp65 deficiency remarkably decreased HBx-related HCC incidence in HBV transgenic mice [37]. In parallel, HBx-induced RAS/RAF/MAPK expressions lead to activation of activating protein-1 (AP-1) and NF-κB transcription factors resulting in disruption of the cell cycle progression [14, 23, 24]. HBx-induced cell cycle progression disruption via upregulation of both p21 and p27 levels, resulting in inhibition of cyclin-dependent kinase (CDK) activity, which in turn enhances MAPK signalling and promotes hepatocytes proliferation [18, 38].

HBx also interacts with host factors p90 ribosomal S6 kinase 2 (RSK2), a member of the 90 kDa ribosomal S6 kinase family that involved in various intracellular processes and cancer pathogenesis. An *in vitro* study found that HBx upregulated the expression of RSK2 in HBV-HCC tissues, HepG2, and SMMC-7721 cells via ERK 1/2 signaling pathway, a member MAPK family. In addition, RSK2 and cyclic adenosine monophosphate (cAMP) response element binding protein (CREB) were both highly expressed in HBV-HCC tissues, and this expression is associated with the increased tumor size [39].

In addition, HBx mutants, particularly C-terminally truncated HBx (ctHBx), also play a multifunctional carcinogenic role in the development of HBV-associated HCC, such as promoting cell cycle progression, increasing cell migration, and regulating the cell cycle and apoptosis. *In vitro* study suggested that ctHBx promoted the formation and development of HCC via cell cycle-related target proteins cell division cycle 25C (cdc25C) and p53 downstream of MAPK pathway [19, 20].

### HBx and oxidative stress

HBx may induce oxidative stress in hepatic cells that further drive carcinogenesis. This is achieved by (1) promotion of lipid peroxidation and reactive oxygen species (ROS) production resulting in abnormal cellular energy metabolisms and (2) downregulation of NADPH quinone oxidoreductase 1 (NQO1) level, an enzyme that detoxifies ROS, indirectly resulting in promotion of glycolysis [25]. In a recent *in vitro* study, hydrogen peroxide was shown to inhibit HBV replication in a p53-dependent fashion by downregulating HBx expression [40]. Recently, the association between protease activated receptor 2 (PAR2) expression and HBx-induced liver injury was also demonstrated. Inhibition of PAR2 expression was shown to suppress HBx-induced inflammation and mitochondrial oxidative stress in hepatic cells, which indicates the potential use of PAR2 antagonist to reduce HBx-induced liver injury [26].

HBx is associated with an abnormal metabolism of energy by raised generation of ROS and consequent oxygen stress-related cell injury. Because of this phenomenon, HBx participates in the endoplasmic reticulum (ER) stress response as well as the respiratory chain in mitochondria, which causes an upregulation of the cytoplasmic ROS levels in hepatocytes. Specifically, based on an HBx-expressing mouse model, elevated oxidative stress in host cells together with a higher expression level of specific inflammatory mediators such as interleukins and TNFs as well as with induction of connected pathways (NF-κB/AKT) [22].

HBx could also influence the level of fatty acid transport protein 2 (FATP2) expression level. FATP2 expression was upregulated by HBx in both *in vitro* and *in vivo* settings, therefore increased FATP2 level may play an important role in the pathogenesis of HBx-induced hepatic steatosis. Indeed, increased FATP2 expression contributed to HBx-induced hepatic lipid accumulation, oxidative stress, and inflammation level resulting in the development of primary HCC. However, reduction of FATP2 level significantly prevents HBx from inducing oxidative stress and inflammation [41].

HBx expression was also linked to the expression of high-mobility group AT-hook 2 (HMGA2), a chromatin-remodeling transcription factor [42]. HMGA2 expression was upregulated in both HCC tissues and cell lines. Silencing of HMGA2 resulted in reduction of oxidative stress in HBx-expressing cells, and correspondingly, this cancer growth promotion effect is diminished together with the suppression of HMGA2 expression [43]. Interestingly, the knockdown of stanniocalcin 2 (STC2), a downstream target of HMGA2, also triggered intrinsic cellular apoptosis [43].

### HBx effect on apoptosis and DNA repair mechanism

The development of HBV-related HCC is closely related to accumulation of DNA damage and replication error. HBV productively infects the liver cell by dysregulating the DNA damage response proteins and consequently, disrupting the intracellular signaling pathways that regulate DNA repair mechanisms [27].

HBx can inhibit cancer cells from apoptosis by increasing the expression of the hepatoma upregulated protein (HURP) and special AT-rich sequence binding protein 1 (*SATB1*) gene, resulting in the overproduction of the anti-apoptotic protein surviving. HBx can also interfere with transcriptional regulation and DNA-binding of p53, plays role as control of apoptosis, cell cycle arrest and DNA repair as previously reviewed [44].

HBx may bind to damaged DNA binding (DDB) protein resulting in disruption of p53 function and induction of cell apoptosis [45–47]. HBx may also inhibit cell cycle control checkpoints and facilitate the accumulation of host mutations through interference of the nt excision repair (NER) mechanism. This effect is achieved by HBx binding to several regulatory proteins that are involved in DNA repair pathways and inhibiting their function [18, 48, 49].

Additionally, HBx may bind to the COOH-terminus of p53, resulting in the relocation of p53 from nucleolus to cytoplasm. This relocation resulted in uncontrolled cell cycle progression and DNA repair mechanism, further promoting the development of HCC [50, 51]. In addition, recently, HBx was reported to suppress the homologous recombination (HR) repair mechanism of the DNA double-strand breaks, through degradation of Smc5/6 complex. This Smc5/6 complex degradation and its associated accumulated DNA damages were observed in both HBV *in vitro* and in *vivo* infection models. Further, this HBx effect on Smc5/6 complex degradation was found reversed by restoring the Smc5/6 complex and maintenance of the HR repair mechanism [28].

### HBx and autophagy mechanism

Autophagy is a major cellular process that breaks down damaged organelles and long-lived proteins for recycling. This process is crucial for maintaining cellular health and homeostasis. However, in the context of cancer, autophagy promotes tumor cell survival, growth, and metastasis.

An *in vitro* study found that HBx induced autophagy within the cells via activation of the PI3K/AKT-mTOR pathway, which leads to an increase expressions of microtubule associated protein 1 light chain 3 beta (LC3B) and beclin 1 (BECN1) proteins, two regulatory proteins involved in formation of autophagosomes [29]. Moreover, HBx enhanced arrestin beta 1 (ARRB1) level in HCC. ARRB1 acts as a crucial coordinator of autophagy through interaction with HBx and LC3B to regulate the formation of autophagosomes and further promotes HBx recruitment of LC3B [30].

HBx also promotes autophagy by downregulating the tumor necrosis factor receptor superfamily member 10b (TNFRSF10B) protein in virus-infected liver cells. HBx inhibits the tumor necrosis factor superfamily member 10 (TNFSF10) receptor signaling via macroautophagy and autophagy-mediated degradation of the TNFRSF10B, one of the TNFSF10 death receptors, resulting in promotion of virus-infected cells survival [52].

Several recent studies further demonstrated the role of HBx in several pathways related to autophagy. In response to Toll-like receptor 4 (TLR4) stimulation, HBx is involved, the retain of the BECN1-Bcl-2 complex and the boost of the TRAF6-BECN1-VPS34 complex, thus enhancing tumor progression [53]. In the NF-κB signaling pathway system, Hbx is able to degrade UBXN7, a member of the ubiquitin regulatory X proteins and a negative regulator of NF-κB. This degradation activates NF-κB signaling and autophagy and affects HBV replication [54].

### HBx and epigenetic modification

The epigenetic modifications mediated by HBx can include DNA methylation, DNA demethylation, histone modifications, chromatin remodeling and the changes in miRNAs profile, altering various cellular functions and contributing to the pathogenesis of HCC [55]. Different from genetic mutations which are permanent, such as HBV gene insertion to the host genome, epigenetic alterations are more dynamic and reversible [56].

Park et al. [31] showed that HBx promotes epigenetic abnormalities, for example, hypermethylation and hypomethylation, that may occur regionally on certain tumor suppressor genes or at the genome-wide scale. This effect is achieved by modulating the function of DNA methyltransferases (DNMTs) immediately after HBV infection and accelerating hepatocarcinogenesis. HBx expression increased total DNMT activities by upregulation of at least three DNMTs variants including DNMT1, DNMT3A1, and DNMT3A2. HBx also induces global hypomethylation of satellite 2 repeat sequences by downregulating expression of DNMT3B [31, 31, 57].

Another study reported that HBx inhibits insulin-like growth factor binding protein-3 (IGFBP-3) production by forming a complex with histone deacetylase 1 (HDAC1). HBx recruits HDAC1 to transcription factor Sp1 and forms HBx-HDAC1 complex that deacetylates Sp1. This complex reduces Sp1 binding ability to DNA and prevents subsequent activation of genes *via* transcriptional repression. By deacetylating Sp1, the HBx-HDAC1 complex silences the *IGFBP-3* gene, resulting in promotion of cell survival, transformation, and cancer progression [32, 51].

The involvement of HBx in histone modification was demonstrated in an HBV replication model, with focus on the histone methyltransferase suppressor of variegation 3-9 homolog 1 (SUV39h1). It induced the methylation of histone H3 lysine 9 (H3K9). The expression of SUV39h1 was increased in liver cancer tissues from rats fed with methyl-deficient diet (MDD) and in patients with HCC. The study demonstrated an interaction between HBx and SUV39h1, contributing to the pathogenesis of HCC [58].

In addition, Chong et al. [59] showed a crucial role for the HBx protein in regulating viral transcription. HBx could control the expression of viral genes by recruiting histone-modifying enzymes (P300 and HDAC), which were essential for transcriptional regulation of the viral minichromosome composed of covalently closed circular DNA (cccDNA) and cellular histones.

miRNAs are small non-coding RNA sequences that take part in various cellular functions including modification of gene expression by binding to the 3’ untranslated region of the target messenger RNA. Changes of various miRNAs expressions have been demonstrated in HCC tissue compared to its non-tumoral counterpart and in sera of HCC patients compared to healthy individuals. In HBV-related HCC, HBx regulation of different miRNAs expressions may influence tumor development by altering the activity of various tumor-related signaling pathways, which in turn can affect the occurrence and progression of cancer.

Dozens of miRNAs have been associated with HBV-related HCC, including for diagnosis, prognosis, and therapeutic applications [60]. A recent microarray study [61], comparing different miRNAs expression among different aetiologies of HCC (HBV, HCV, and non-HBV non-HCV), showed that only miR-210-3p expression was significantly increased in the HBV-related HCC tissue samples. In addition, miR-210-3p also regulated HBx expression and miR-210-3p inhibition could prevent hepatocarcinogenesis due to HBV infection [61].

Other important recent *in vitro* and *in vivo* experiments had identified several miRNAs related to HBx. miR-187-5p expression contributed to HBx-induced HCC progression through E2F1/FoxP3 signaling pathway [33]. Dysregulated HBx expression also increases the expressions of miR-21 [62], miR-29, miR-155 [63, 64], miR-221/222 [65] and miR-520c-3p [66], resulting in inhibition of the expression of phosphatase and tensin homolog on chromosome 10 (PTEN). By suppressing PTEN expression, HBx promotes the AKT/mTOR signaling, leading to cancer cell growth and proliferation [67]. While, HBx downregulated the expression of let-7c and miR-99a, it also enhanced the expression of an enhancer of zeste homolog 2 (EZH2) [68].

**Figure 1:**
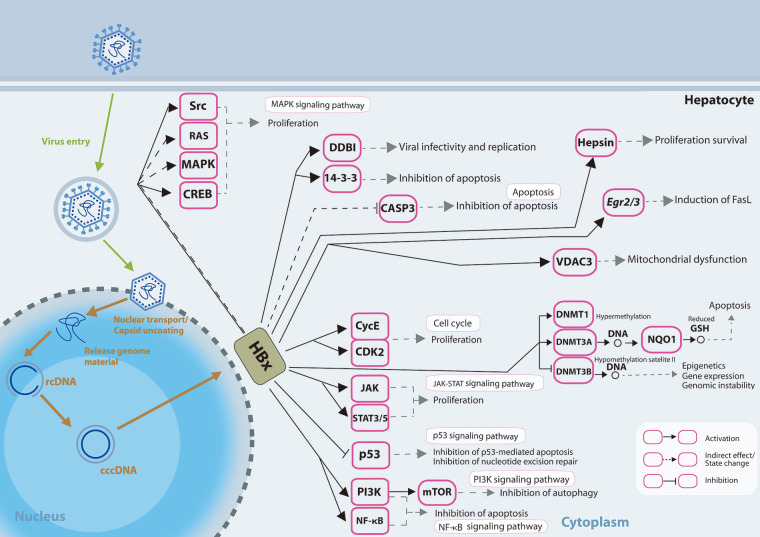
HBx involvement in promoting hepatocarcinogenesis. HBx protein operates on numerous receptors, transcription factors, and components of the transcription machinery, thus dysregulating various cellular signalling pathways and epigenetic components. rcDNA: relaxed circular DNA; CASP3: caspase-3; *Egr2/3*: early growth response gene 2/3; VDAC3: voltage-dependent anion chanel 3; CycE: cyclin E; STAT3/5: signal transducer and activator of transcription 3/5; GSH: glutathione; FasL: Fas ligand

Different mechanisms on how HBx induced dysregulated miRNA expressions may affect and are affected by various epigenetic modifications, and how the changes may result in further modulation of viral and host genome expressions has been reviewed previously [69]. The presentation of how HBx affects the signalling pathways in the liver cells is shown in Figure 1.

### HBV X gene insertion in the human genome

Next generation sequencing on HBV-mediated HCC samples had identified that over 80% of HBV-positive cases have HBV genome integration into the host genome [70]. Three cancer-associated genes have been identified as the most frequent integration sites in HBV-associated tumors, including telomerase reverse transcriptase (TERT), epigenetic regulator MLL4, and cell cycle gene *CCNE1*. Further, analysis of the integration breakpoint sites demonstrated that 40% of these breakpoints were localized to a particular 1,800 bp region of the HBV genome that encompasses viral enhancer, *X* gene, and part of the core region [71, 72].

HBV gene integration into hepatocytes can cause genetic instability and lead to tumorigenesis through several mechanisms. First, HBV binds to UV-DDB, a protein involved in DNA repair and S-phase progression [73], leading to mutations, insertions, deletions, and rearrangements of the host genome [74]. Secondly, integration of HBV DNA may result in cis-activation of various oncogenes or altered activation of the tumor suppressor genes. HBV integration resulted in dysregulation of various genes involved in cell growth and proliferation regulation, leading to uncontrolled cell growth and promotion of tumor formation. Lastly, integration of the HBV DNA may affect the production of mutant HBV proteins with different tumor-promoting potential [71].

A recent study [75] had reported that a higher number of HBV integration was more frequent in non-HCC tumour tissues, and that integration of the viral genomes resulted in promotion of both local and distant oncogenic driver expression in liver cancer cells. In addition, the number of HBV integrations may be used as prognostic factor for HBV-related HCC, since the number of integrations was associated with poor disease prognosis [75].

### *HBx* gene mutation

There is an overlap between the HBx gene and the HBV core promoter region, which is crucial for viral life cycle [76]. The A1762T/G1764A double mutations in X gene or core promoter has been correlated with advanced liver disease. These mutations were reported as a predisposition factor for HCC and can be detected in hepatitis B patient around 10 years before HCC is even detected [77, 78]. Another study has also reported that this double mutation is associated with HCC progression [79]. In the last two decades, the frequencies of A1762T/G1764A double mutations in chronic HBV patients have become more dominant, with higher frequencies of these mutants in cirrhosis and HCC patient groups [80, 81]. Another study on HBV genotype C, further demonstrated that this double mutation A1762T/G1764A is positively with worse-disease progression and development of HCC, even in the absence of cirrhosis [82].

The A1762T/G1764A double mutation resulted in changes in HBx AA sequence, changing the codons 130 and 131 of the HBx protein, from lysine (K) to methionine (M) and from valine (V) to isoleucine (I), respectively. The K130M/V131I mutations enhanced the HBx-induced effect on hypoxia-inducible factor-1a (HIF-1a). This upregulated expression and transcriptional activity of HIF-1a facilitates cancer cells survival and proliferation by promoting angiogenesis [83]. In addition, K130M/V131I mutations also lead to an increase S-phase kinase-associated protein 2 (SKP2) production but a decrease cyclin kinase inhibitor p21 production. These changes may promote the inhibition of precore mRNA and increase the transcription of pregenomic RNA (pgRNA) resulting in increased viral replication and liver carcinogenesis [78, 84].

In addition, other HBx mutations, such as A1630G, G1721A, A1762T, G1764A and A1774G mutations, have also been reported to be involved in the genome integration process into hepatocyte chromosome and as such correlated to hepatocarcinogenesis [85]. These observations indicate that HBV genetic variation might influence to the development of HCC.

### HBx as modulator of long non-coding RNA and circular RNA

Long non-coding RNA (lncRNA) and circular RNA (circRNA) are newly recognized non-coding RNAs (ncRNAs) that have recently gained attention. Both lncRNA and circRNA have shown substantial roles in HCC pathogenesis and due to their stability, these ncRNAs are even proposed as potential biomarkers for diagnostic and disease progression [86]. LncRNA and circRNA have the potential to function as regulators of the liver microenvironment and chronic liver diseases including viral infection, transcriptional activation through DNA and protein binding, protein translation, as well as serving as sponges for miRNAs and mRNAs [87, 88].

As a regulatory protein, HBx regulates the activity of a variety of coding genes and ncRNA promoters including lncRNA and circRNA. Several studies have demonstrated the link between HBx and ncRNAs specifically in HCC.

HBx boosts transcription and prompts the accumulation of deleted in lymphocytic leukemia 2 (DLEU2), an lncRNA expressed in the liver, and increased in HCC. Together, HBx and DLEU2 regulate transcription of the cccDNA minichromosome of HBV, thereby enhancing HBV replication. HBx-DLEU2 association also affects host epigenetic control by increasing EZH2 expression in HBV-replicating cells and in HBV-related HCCs [89]. Moreover, the interaction of DLEU2 and EZH2 also enhances the proliferation, migration, and invasion capabilities of HCC cells, thereby intensifying the progression of HCC [90]. In another study, HBx also enhances the expression of lncRNA urothelial cancer associated 1 (UCA1) and fosters cell proliferation and the development of tumors by attracting EZH2 and suppressing p27Kip1/CDK2 signalling [91].

The progression of HBx-related HCC is also closely linked to the contribution of ncRNA miR-124 and lncRNA metastasis-associated lung adenocarcinoma transcript 1 (MALAT1). Enhanced expression of miR-124 or suppression of lncRNA-MALAT1 prevents HBx-triggered cancer stem cell formation, activation of stemness-related factors, and tumorigenicity by inhibiting the PI3K/AKT signaling pathway [92].

LINC01010 is another lncRNA affected by HBx, notably decreased in both HepG2-4D14 cells and liver tissues of HCC patients, and it exhibits a positive correlation with survival. It was observed that the HBV-encoded HBx can diminish the transcription of LINC01010. Functional analysis revealed that increasing the levels of LINC01010 impedes the proliferation, migration, and invasion of HepG2 cells, whereas reducing LINC01010 enhances these processes [93].

HBx also modulates circRNA expression in HCC. HBx can induce the degradation of cFAM210A, a circRNA originating from the *FAM210A* gene, influencing the proliferation, stemness, and tumorigenicity of HCC cells. The downregulation of cFAM210A expression has a negative correlation with tumorigenesis in individuals with HBV-related HCC [94]. HBx can reduce circSFMBT, a tumor suppressor circRNA, and facilitate the HCC metastasis through the miR-665/TIMP3 axis [88]. The aforementioned studies highlight that HBx plays a role in modulating both the oncogenic and tumor suppressor ncRNAs.

## Conclusions

HBx as a multifunctional regulatory protein interfering with several signaling pathways that drive HBV-associated hepatocarcinogenesis. HBx expression may induce and/or support the progression of HCC since it can modulate the liver cellular microenvironment by affecting various cell regulatory factors and mechanisms including transcription factors, cell cycle regulators, apoptosis and DNA repair mechanisms, autophagy, and epigenetic mechanisms. Recently, HBx is also associated in modulating both the oncogenic and tumor suppressor ncRNAs. In addition, some mutations found in HBx region are also associated with HCC development and progression since these mutations increased viral replications, enhanced survival, and proliferation of cancerous cells.

## Abbreviations

AA: amino acid
AFP: α-fetoprotein
AKT: protein kinase B
BECN1: beclin 1
cccDNA: covalently closed circular DNA
CDK: cyclin-dependent kinase
DDB: damaged DNA binding
DLEU2: deleted in lymphocotic leukemia 2
DNMTs: DNA methyltransferases
EZH2: enhancer of zeste homolog 2
FATP2: fatty acid transport protein 2
HBV: hepatitis B virus
HBx: hepatitis B virus X protein
HCC: hepatocellular carcinoma
HCV: hepatitis C virus
HDAC1: histone deacetylase 1
HMGA2: high-mobility group AT-hook 2
JAK-STAT: Janus kinase-signal transducer and activator of transcription
LC3B: light chain 3 beta
lncRNA: long non-coding RNA
MAPK: mitogen-activated protein kinase
miRNAs: microRNAs
mTOR: mammalian target of rapamycin
ncRNAs: non-coding RNAs
NF-κB: nuclear factor kappa-light-chain-enhancer of activated B cells
nt: nucleotide
ORFs: open reading frames
PAR2: protease activated receptor 2
PI3K: phosphatidylinositol 3-kinase
RAS: rat sarcoma virus
ROS: reactive oxygen species
RSK2: ribosomal S6 kinase 2
SUV39h1: suppressor of variegation 3-9 homolog 1

## Declarations

### Copyright

© The Author(s) 2024.
